# Activation of autophagy attenuates EtOH-LPS-induced hepatic steatosis and injury through MD2 associated TLR4 signaling

**DOI:** 10.1038/s41598-017-09045-z

**Published:** 2017-08-24

**Authors:** Xiaoxia Kong, Ying Yang, Li Ren, Tuo Shao, Fengyuan Li, Cuiqing Zhao, Liming Liu, Hongyu Zhang, Craig J. McClain, Wenke Feng

**Affiliations:** 1 School of Basic Medical Sciences, Wenzhou Medical University, Wenzhou, Zhejiang China; 2 School of Pharmaceutical Sciences, Wenzhou Medical University, Wenzhou, Zhejiang China; 3 1st Affiliate Hospital, Xi’an Jiaotong University, Xi’an, China; 4 Departments of Pharmacology and Toxicology and Medicine, University of Louisville Alcohol Research Center, University of Louisville Hepatobiology & Toxicology Program, University of Louisville, Louisville, USA; 5 Institute of Virology, Wenzhou University, Louisville, KY USA; 6 2nd Affiliate Hospital, Wenzhou Medical University, Louisville, KY USA; 7 Robley Rex Louisville VAMC, Louisville, KY USA

## Abstract

Autophagy serves as a protective mechanism to degrade damaged organelles and proteins. Acute alcohol exposure is known to activate the hepatic autophagy response, whereas chronic alcohol exposure slows autophagosome formation along with an elevation of gut-derived endotoxin. In the current study, we examined whether lipopolysaccharide (LPS) administration decreased autophagic response in the liver of mice treated by short-term alcohol and whether activation of autophagy by rapamycin attenuates EtOH-LPS-induced liver steatosis and injury. We demonstrated that ten-day alcohol feeding primed the liver to LPS-induced lipid accumulation and liver injury with significantly increased hepatic steatosis and serum AST level as well as hepatic cellular NF-κB activation. LPS increased alcohol-mediated reactive oxygen species (ROS) formation while reducing autophagy activation. These deleterious effects were attenuated by rapamycin administration in mice. The protective effects of rapamycin are associated with decreased cellular MD2/TLR4 expression and interaction in Raw264.7 cells. Taken together, our results demonstrated that enhanced gut-derived LPS decreases the hepatic autophagosome numbers in response to alcohol exposure, and activation of autophagy by rapamycin protects from EtOH-LPS-induced liver injury, probably through reduced macrophage expression and interaction of TLR4/MD2 signaling complex.

## Introduction

Alcoholic liver disease (ALD) is one of the major causes of liver-related morbidity and mortality worldwide^1^. Excessive alcohol consumption induces hepatic steatosis, which can lead to advanced liver diseases, such as steatohepatitis, fibrosis, cirrhosis and potentially hepatocellular carcinoma (HCC). Hepatic steatosis as a result of alcohol consumption is initially benign and can be resolved by abstinence. However, it sensitizes the liver to injury by subsequent insults. This “two-hit hypothesis” suggests that alcohol alone is not solely responsible for the progression to ALD from steatosis; rather, a second insult is required for the development of simple steatosis to steatohepatitis, fibrosis and cirrhosis. Metabolism of alcohol in the gut and liver increases reactive oxygen species (ROS), changes gut microbiota and increases lipopolysaccharide (LPS) production leading to an increased inflammatory response which results in a deleterious feedforward loop that exacerbate liver inflammation and lipid dysmetabolism. Clinical studies have provided considerable evidence that LPS is in the serum and liver of the patients with ALD^2^. LPS released from the gut binds to toll-like receptor 4 (TLR4) and myeloid differentiation protein 2 (MD2) on the surface of Kupffer cells^3^ and activates NF-κB-mediated proinflammatory pathways^4, 5^. Consistent with this hypothesis, Kupffer cells from alcohol-fed mice exhibit increased LPS responses, leading to higher levels of TNFα and MCP-1 production^6^. However, the precise mechanisms underlying the role of LPS-alcohol interaction in ALD are still not fully understood.

Macroautophagy (hereafter autophagy) is a lysosomal pathway that degrades superfluous or damaged organelles. These cytoplasmic cargos are trapped inside autophagosomes that ultimately fuse with lysosomes for degradation of their contents^7–9^. Subsequently, the breakdown products are released into the cytosol and contribute to energy and metabolic supply, particularly in starvation. Recent studies reported that autophagy is involved in the adaptation to alcoholic liver injury^10, 11^. Autophagy can either be increased or decreased by ethanol depending on the model used, the dose, the tissue evaluated and the experimental conditions^12–15^. Acute ethanol consumption elevates autophagy, and may serve as a protective mechanism against ethanol toxicity, and inhibition of autophagy increases ethanol toxicity and steatosis^16^.

The current study aimed to further determine the role of autophagy in ALD in mice with chronic alcohol exposure and subsequent LPS challenge. We found that LPS insults decrease the protective effect of EtOH-induced autophagy, and activation of autophagy by rapamycin protects the liver from EtOH-LPS-induced injury through decreased MD2 associated TLR4 activation.

## Results

### Alcohol exposure sensitizes liver to LPS-induced steatohepatitis

**Figure 1:**
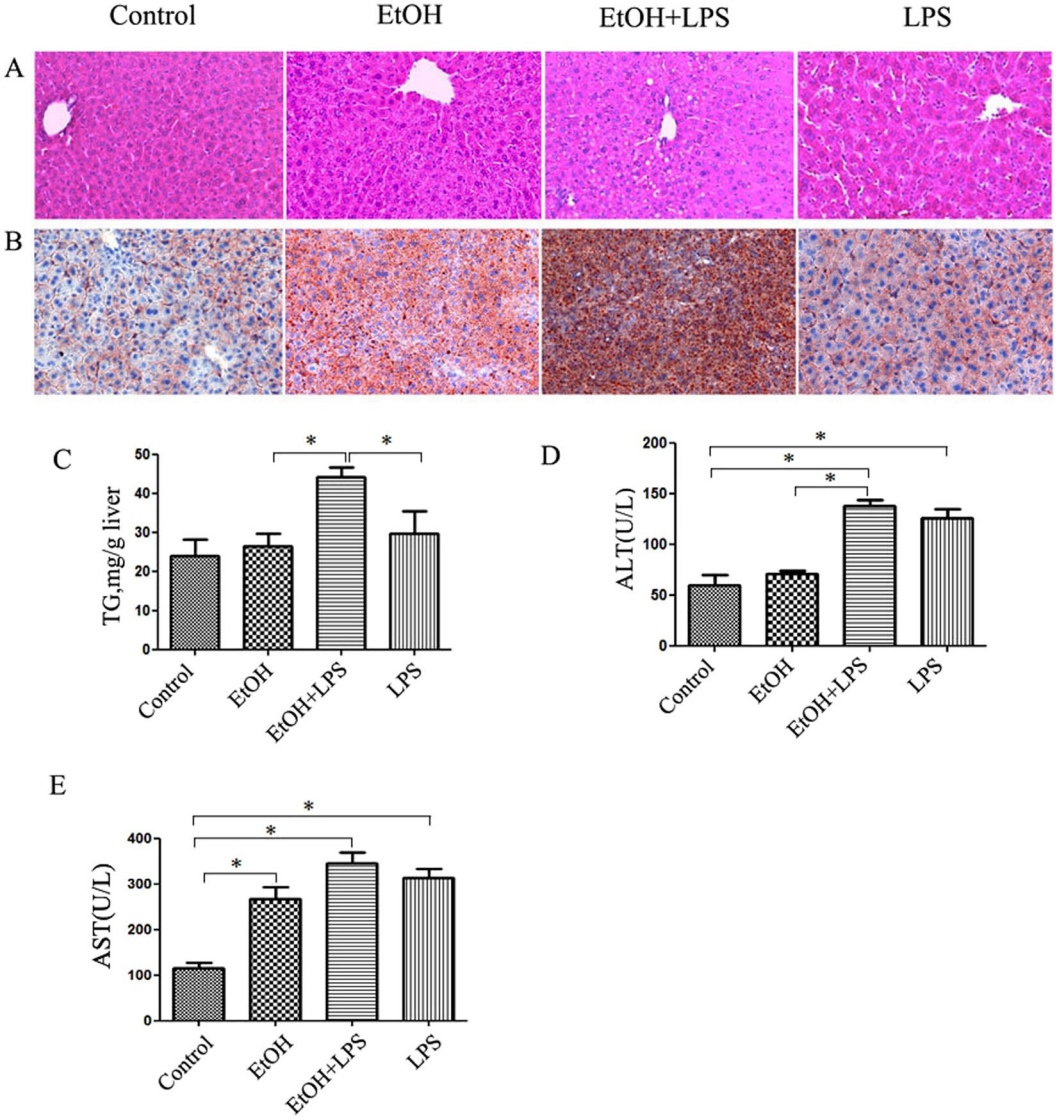
LPS aggravates alcohol-induced hepatic steatosis and liver injury. Male C57BL6 mice were fed a Lieber DeCarli liquid diet containing 5% ethanol for 10 days followed by an LPS injection at a dose of 10 mg/kg i.p. Six hours later, the mice were sacrificed for analysis. (**A**) H&E staining of the liver sections. (**B**) Hepatic fat levels were evaluated by Oil red O staining of frozen liver tissue sections. (**C**) Hepatic tissue triglyceride (TG) levels. Serum levels of ALT (**D**) and AST (**E**). Results are presented as means ± SEM. *p < 0.05. Abbreviation: ALT, alanine transaminase; AST, aspartate transaminase.

Hepatic steatosis was evaluated with histological analysis and triglyceride measurement. As shown in Fig. 1, alcohol or LPS alone produced an insignificant increase in hepatic lipid accumulation. However, LPS injection significantly increased hepatic fat content in mice fed alcohol compared to the mice fed control diet, indicating a sensitization of liver by alcohol to LPS insult in terms of lipid accumulation (Fig. 1A,B). Confirming the histology findings, liver tissue triglyceride content was significantly higher in LPS-EtOH mice than in control mice and LPS mice group (Fig. 1C). These data suggest that alcohol feeding obviously increased liver tissue triglyceride content in LPS mice. To assess liver injury induced by alcohol and LPS, plasma ALT and AST activities were determined. Serum ALT activity was moderately while AST level was significantly increased by alcohol feeding. LPS injection markedly increased both ALT and AST levels, and interestingly, both liver enzymes were higher in alcohol-exposed mice compared to pair-fed (PF) mice upon LPS challenge, but there was no significantly difference between LPS-EtOH group and LPS group (Fig. 1D,E). Taken together, our results suggest that 10-day alcohol feeding sensitizes liver to LPS-induced lipid accumulation and liver injury.

### Alcohol pre-exposure exacerbates LPS-induced reactive oxygen species (ROS) formation and NF-κB activation

**Figure 2:**
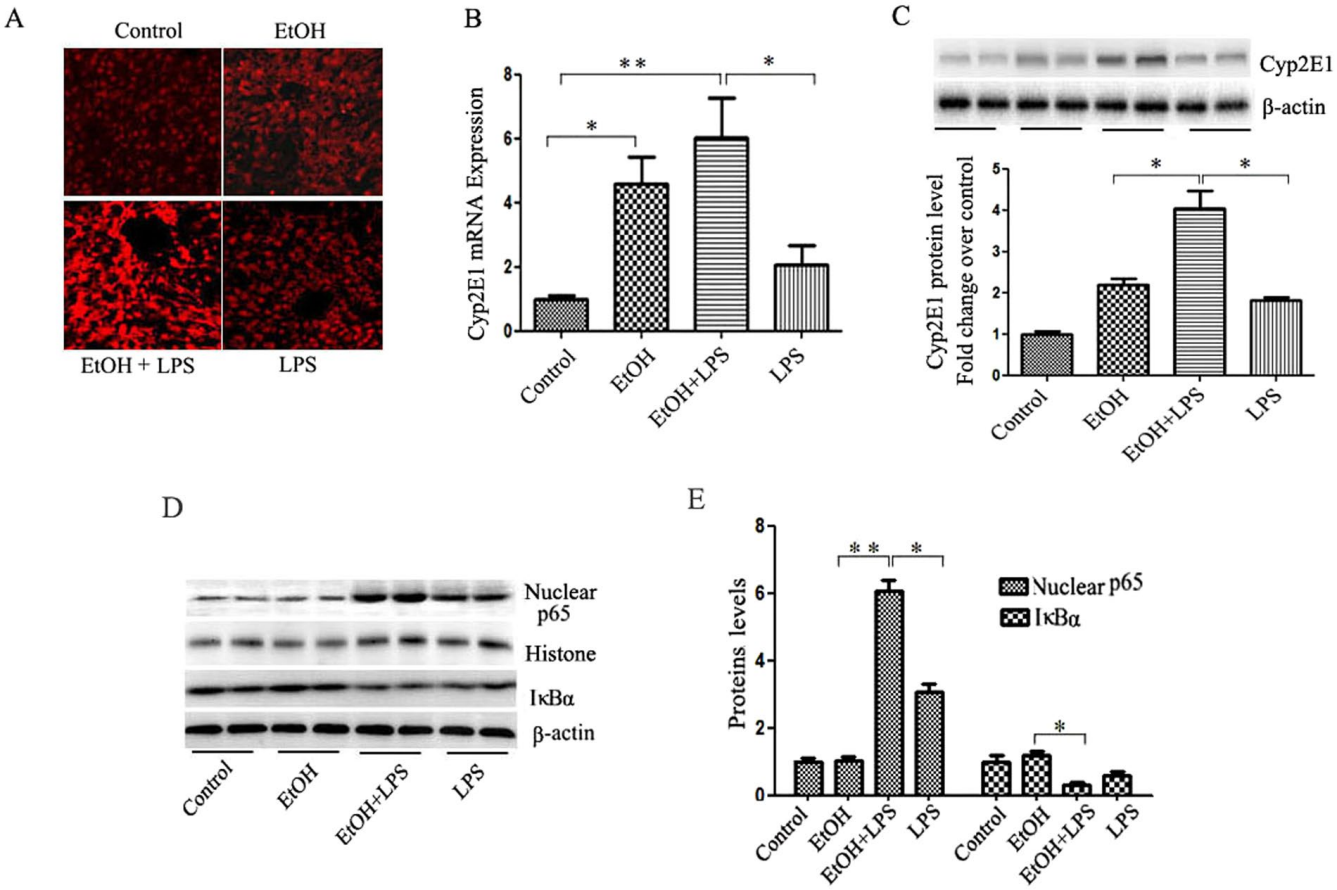
Alcohol increases LPS-induced ROS formation and NF-κB signaling. (**A**) The superoxide anion-catalyzed ethidium red fluorescence was examined using confocal microscopy (400 × magnification). (**B**) Liver Cyp2E1 mRNA levels were analyzed by real-time RT-PCR. (**C**) Expression of Cyp2E1 protein was detected by Western blotting, the ratios of Cyp2E1 to β-actin. (**D**) Nuclear NF-κB p65 expression and IκBα protein expression in the total hepatic fractions. Histone and β-actin was used as loading control. (**E**) Quantification analysis of the results. Data are expressed as mean ± SEM (n = 6). *P < 0.05, **P < 0.01. Abbreviation: DHE, dihydroethidium; Cyp2E1, cytochromeP450.

Alcohol-induced steatosis and liver injury are attributed the increased ROS formation. Ethanol metabolism in the liver involves cytochrome P4502E1 (Cyp2E1), which is known as a major mechanism in generating ROS^17, 18^. To determine whether oxidative stress is involved in the liver sensitization by alcohol and LPS insult, we evaluated ROS production by DHE staining of frozen sections of liver tissues. Ten-day alcohol feeding generated higher superoxide staining in liver compared to pair feeding. However, one dose of LPS injection increased ROS production, although not statistically significant. Interestingly, when mice were pre-exposed to alcohol, LPS injection induced a marked elevation in ROS production (Fig. 2A). Next, we determined hepatic Cyp2E1 expression in mRNA and protein levels. As shown in Fig. 2B,C, alcohol feeding significantly increased Cyp2E1 gene expression and protein concentration compared to control diet feeding, while LPS alone did not produce significant elevation in both mRNA and protein levels.

LPS stimulates pro-inflammatory cytokine production through NF-κB signaling, which plays a critical role in the development and progression of ALD. In the canonical NF-κB pathway, NF-κB activation depends on IκBα phosphorylation and degradation. We thus examined the effect of alcohol on the expression of active NF-κB p65 and IκBα protein in liver tissues. As shown in Fig. 2D,E, LPS injection induced a moderate increase in nuclear NF-κB protein levels in the liver, while alcohol pre-exposure dramatically increased LPS-induced NF-κB p65 expression more than 6 folds. In addition, hepatic IκBα protein levels were significantly decreased in EtOH-LPS group. These results indicated that alcohol pre-exposure increased LPS-induced formation of ROS and activation of NF-κB signaling.

### LPS treatment reduces autophagy activation by alcohol

**Figure 3:**
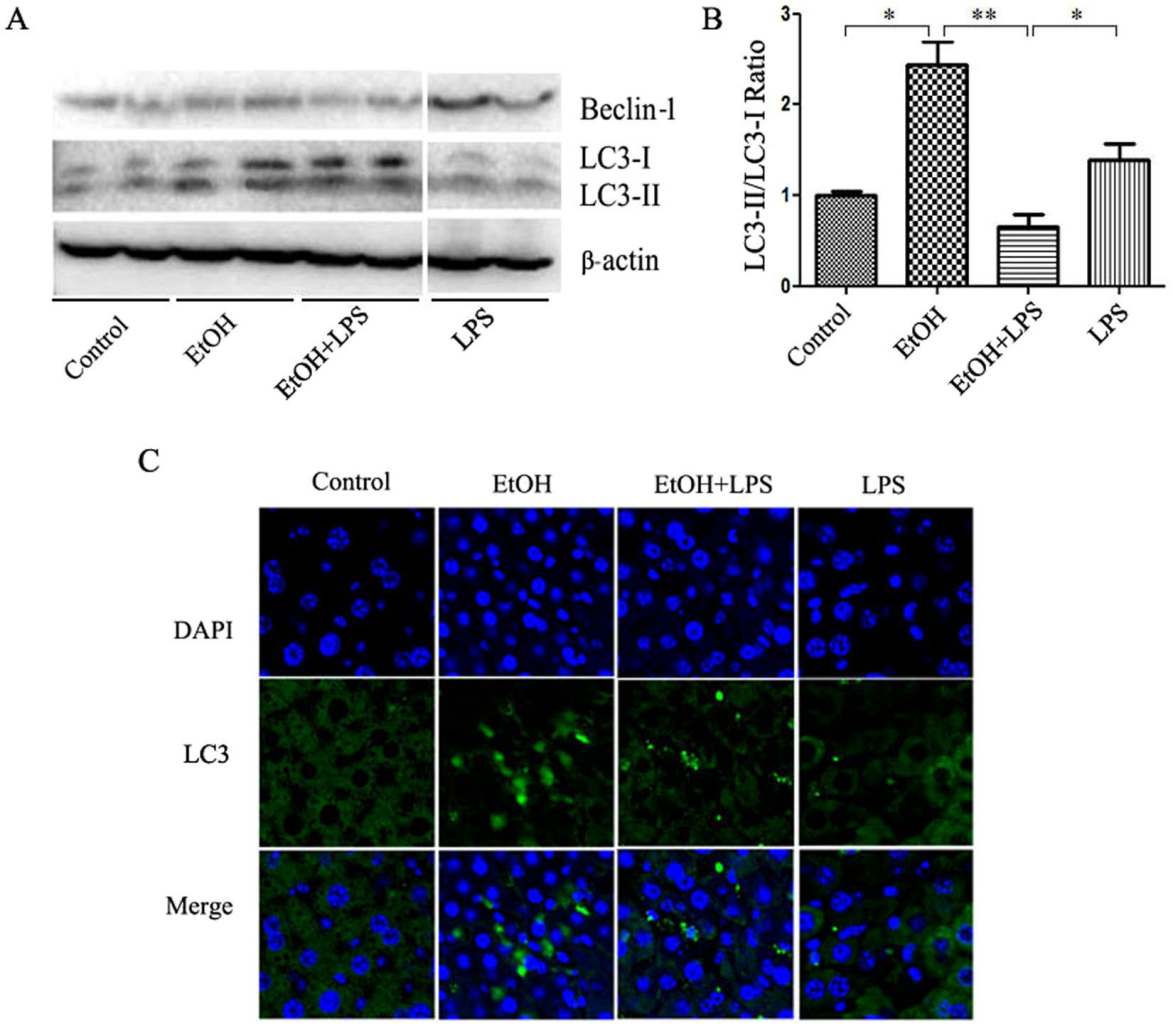
LPS damages autophagy activation by alcohol in the liver. (**A**) LC3 and Beclin-1 protein levels determined by Western blotting, and β-actin was used as loading control. Two lanes between ETOH + LPS group and LPS group were chopped due to unrelated to this figure. The full blot was placed in the Supplementary Information (Supplementary Figure S4). (**B**) LC3 expression was quantified by densitometry analysis and normalized to β-actin expression. (**C**) Immunofluorescent analysis of LC3 (green). Images are at 800× magnification. Data are expressed as mean ± SEM, (n = 6). *p < 0.05, **p < 0.01. Abbreviation: LC3, microtubules associated protein 1 light chain 3β.

Studies indicate that autophagy can either be increased or decreased by ethanol. To determine the changes in autophagy by alcohol and LPS insults, we analyzed Beclin-1 and LC-3 protein levels by Western blot and immunofluorescent staining of liver tissues. As shown in Fig. 3A,B, alcohol pre-exposure increased the expression of autophagic proteins, LC3-II and Beclin-1, in the liver. LPS injection alone increased Beclin-1 protein levels but not LC-3 activation. Importantly, LPS injection markedly reduced the alcohol-induced autophagic response, as seen in the LC3-II/LC3-I ratio change. Furthermore, LC3 immunofluorescence staining analysis showed an extensive induction of the formation of LC3 puncta, representing autophagosome formation, in mice fed alcohol for 10 days (Fig. 3C). However, the autopagic induction was reduced by LPS injection. These results indicated that LPS insult decreased the autophagic response in the liver as a result of pre-exposure to alcohol.

### Rapamycin decreases EtOH-LPS-induced liver injury

**Figure 4:**
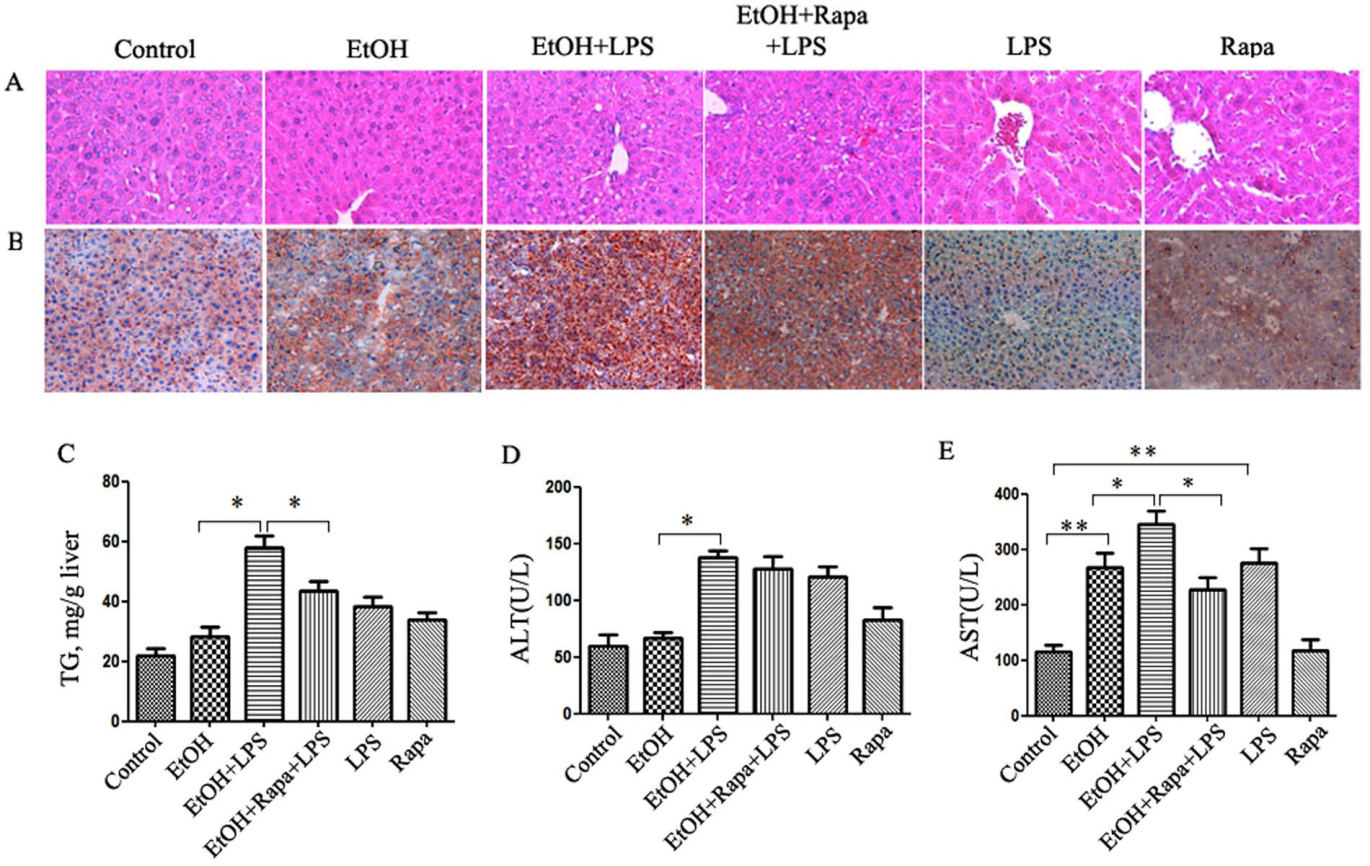
Autophagy activation by rapamycin decreases EtOH-LPS-induced hepatic steatosis and injury. (**A**) H&E staining of liver sections. (**B**) Oil red O staining of frozen tissue sections. (**C**) Liver tissue triglyceride (TG) levels. Serum liver ALT (**D**) and AST (**E**) levels. Data are expressed as mean ± SEM (n = 6). *p < 0.05. Abbreviation: ALT, alanine transaminase; AST, aspartate transaminase.

Since LPS reduces the autophagic response, activation of autophagy pharmacologically should protect liver from EtOH-LPS-induced injury. Rapamycin, an autophagy inducer, was administered 24 hours before LPS injection to the mice exposed to alcohol. As expected, rapamycin markedly increased the formation of LC3 puncta even under the conditions of EtOH-LPS treatment (Supplementary Figure S1A). Protein analysis by Western blotting showed that the reduction of LC3-II/LC3-I ratio by LPS in alcohol pre-exposed mice was inhibited by rapamycin (Supplementary Figure S1B and C). We then determined the protective role of the autophagy induction by rapamycin in the liver injury induced by EtOH-LPS. As shown in Fig. 4, rapamycin treatment improved hepatic histology and steatosis (Fig. 4A,B). Confirming the histology observation, rapamycin significantly decreased the elevated hepatic triglyceride content induced by EtOH-LPS (Fig. 4C). In addition, we found that there was a significant reduction in AST level as a result of rapamycin administration (Fig. 4D,E). Taken together, activation of autophagy by rapamycin significantly improved EtOH-LPS-induced hepatic steatosis and liver injury.

Induction of oxidative stress is an important factor for ALD development and progression^19^. Recent studies have shown that autophagy is protective in acute alcohol/Cyp2E1-dependent liver injury^20^. Next, to determine whether the protective effect of rapamycin in EtOH-LPS-induced liver injury involves Cyp2E1-dependent oxidative stress, the ROS levels were measured in frozen sections of liver tissue by immunofluorescence staining. As expected, rapamycin-induced autophagic activation significantly lowered ROS production (Supplementary Figure S2A). Liver Cyp2E1 mRNA level was increased significantly by EtOH-LPS exposure, which was attenuated by rapamycin administration (Supplementary Figure S2B). These results imply that rapamycin decreased EtOH-LPS-induced ROS formation.

### Induction of autophagy by rapamycin decreases inflammatory cytokine production via inhibiting MD2/TLR4 expression

**Figure 5:**
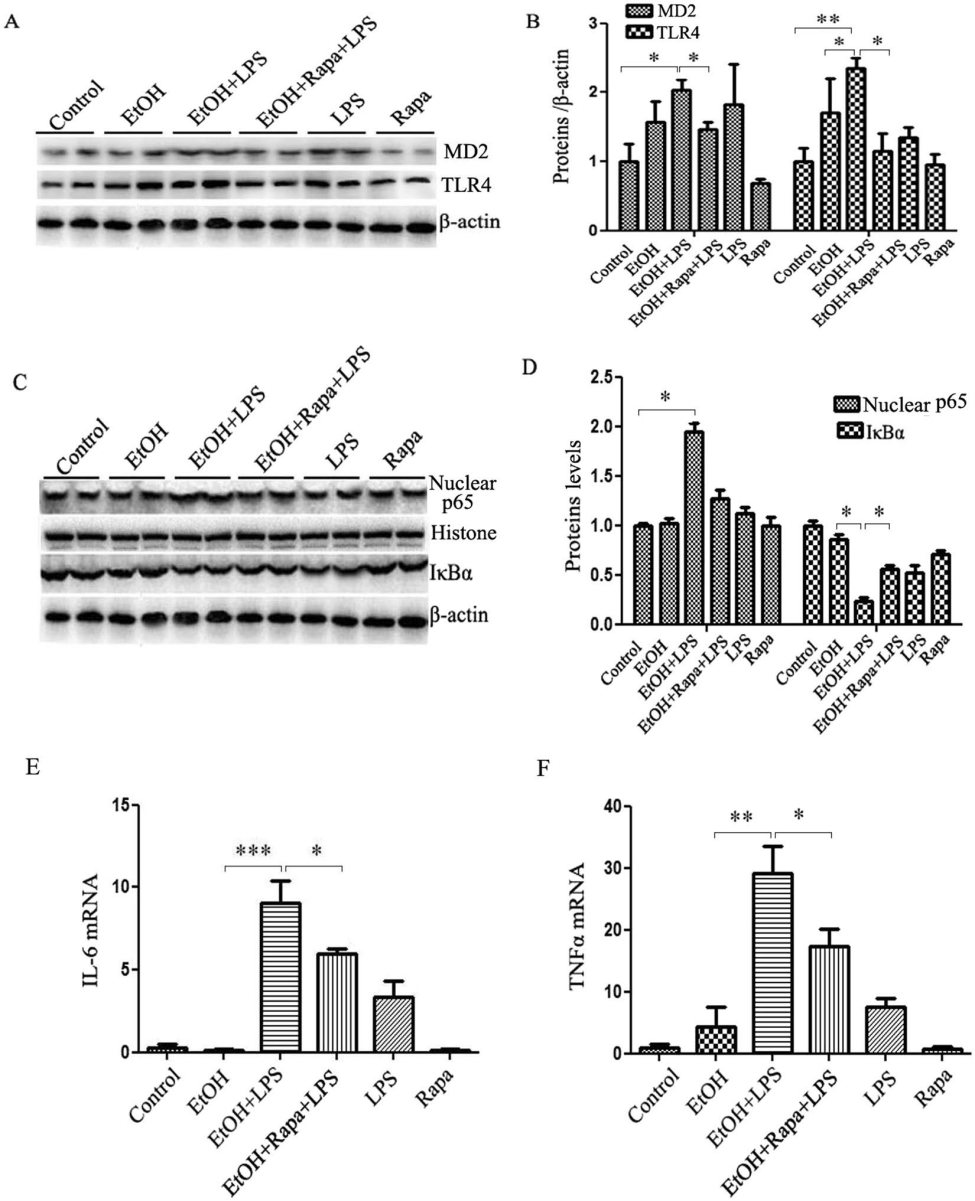
Induction of autophagy by rapamycin decreases inflammatory cytokine production by reducing hepatic MD2/TLR4 expression. (**A**) Expression of MD2 and TLR4 proteins in the liver tissue. (**B**) Quantified results of data in A and normalized to β-actin levels. (**C**) Expression of NF-κB p65 and IκBα in the liver tissues. (**D**) Quantification analysis of the results. Liver IL-6 (**E**) and TNFα (**F**) mRNA levels were analyzed by real-time RT-PCR. Data are expressed as mean ± SEM (n = 6). *p < 0.05, **p < 0.01, ***p < 0.001. Abbreviation: MD-2, myeloid differentiation 2; TLR, toll like receptor; NF-κB, nuclear factor κB; TNFα, tumor necrosis factor α.

LPS activates an inflammatory response through TLR4 signaling. To determine the role of rapamycin in the attenuation of EtOH-LPS-induced liver injury through TLR4-mediated signaling, we analyzed hepatic protein levels of TLR4 and its activation complex partner, MD2. As shown in Fig. 5A,B, both TLR4 and MD2 protein levels were slightly increased by alcohol or LPS alone, but significantly elevated by LPS in alcohol pre-exposed mouse livers. Importantly, rapamycin administration markedly inhibited these inductions. As a consequence, the downstream targets of TLR4/MD2 were inhibited, as shown in Fig. 5C,D for nuclear NF-κB p65 and IκB protein levels. Hepatic IL-6 and TNF-α mRNA expression was increased dramatically by LPS in alcohol pre-exposed livers, and that expression was significantly reduced by rapamycin treatment (Fig. 5E,F). These results suggest that induction of autophagy by rapamycin inhibits MD2/TLR4 signaling pathways resulting in a decreased hepatic NF-κB level and pro-inflammatory cytokine production in EtOH-LPS exposed mice.

### Rapamycin inhibits LPS-attenuated autophagic response in alcohol incubated Raw264.7 cells

**Figure 6:**
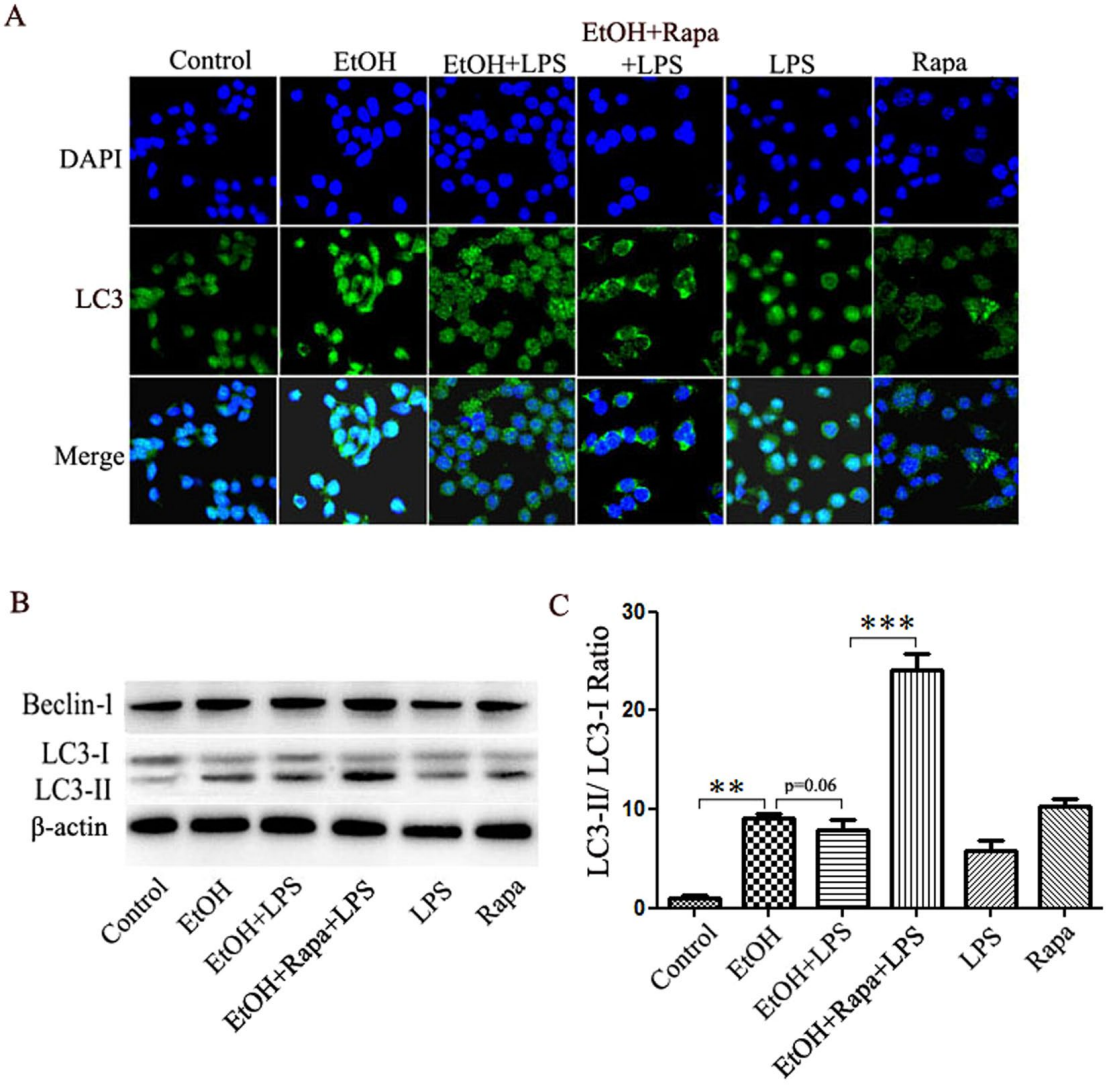
Rapamycin activates autophagy in the Raw264.7 cells treated by EtOH + LPS. (**A**) Raw264.7 cells were labeled with anti-LC3 antibody, and the LC3-II puncta were recorded by confocal microscopy. Number of LC3-II puncta in each cell was counted over 100 cells in each group. (**B**) LC3 and Beclin-1 protein levels. (**C**) LC3 levels were quantified by densitometry analysis and normalized to β-actin levles. Images are at 800× magnification. Data are expressed as mean ± SEM (n = 5). **p < 0.01, ***p < 0.001. Abbreviation: LC3, microtubules associated protein 1 light chain 3β.

TLR4 and MD2 are highly expressed in hepatic Kupffer cells, the resident hepatic macrophage cells. To determine whether macrophage cells are responsible for the protective effects of the autophagy induction by rapamycin in EtOH-LPS-induced liver injury, we exposed Raw264.7 cells to alcohol (50 mM) for 48 hours followed by 6 hours incubation with LPS (100 ng/mL). Rapamycin was added at a dose of 50 nmol/L for 24 hours before LPS treatment. LC3 immunofluorescence staining showed an elevation of autophagosome by alcohol incubation, which was decreased by LPS treatment. Importantly, rapamycin inhibited LPS-diminished autophagic response (Fig. 6A). The activation of autophagy was also determined by Western blot analysis of autophagy markers. The expression of LC3-II increased significantly by alcohol treatment and slightly decreased by LPS treatment. However, rapamycin treatment dramatically increased the LC3-II/LC3-I ratio by more than 2-fold in EtOH-LPS treated Raw264.7 cells (Fig. 6B,C). Another autophagy marker, Beclin-1 had a similar response (Fig. 6B). Furthermore, rapamycin decreased ROS formation in Raw264.7 cells (Supplementary Figure S3), a beneficial effect similar to what we found in the *in vivo* study above.

### TLR4/MD2 is involved in the inhibition of inflammation by rapamycin in Raw264.7 cells

**Figure 7:**
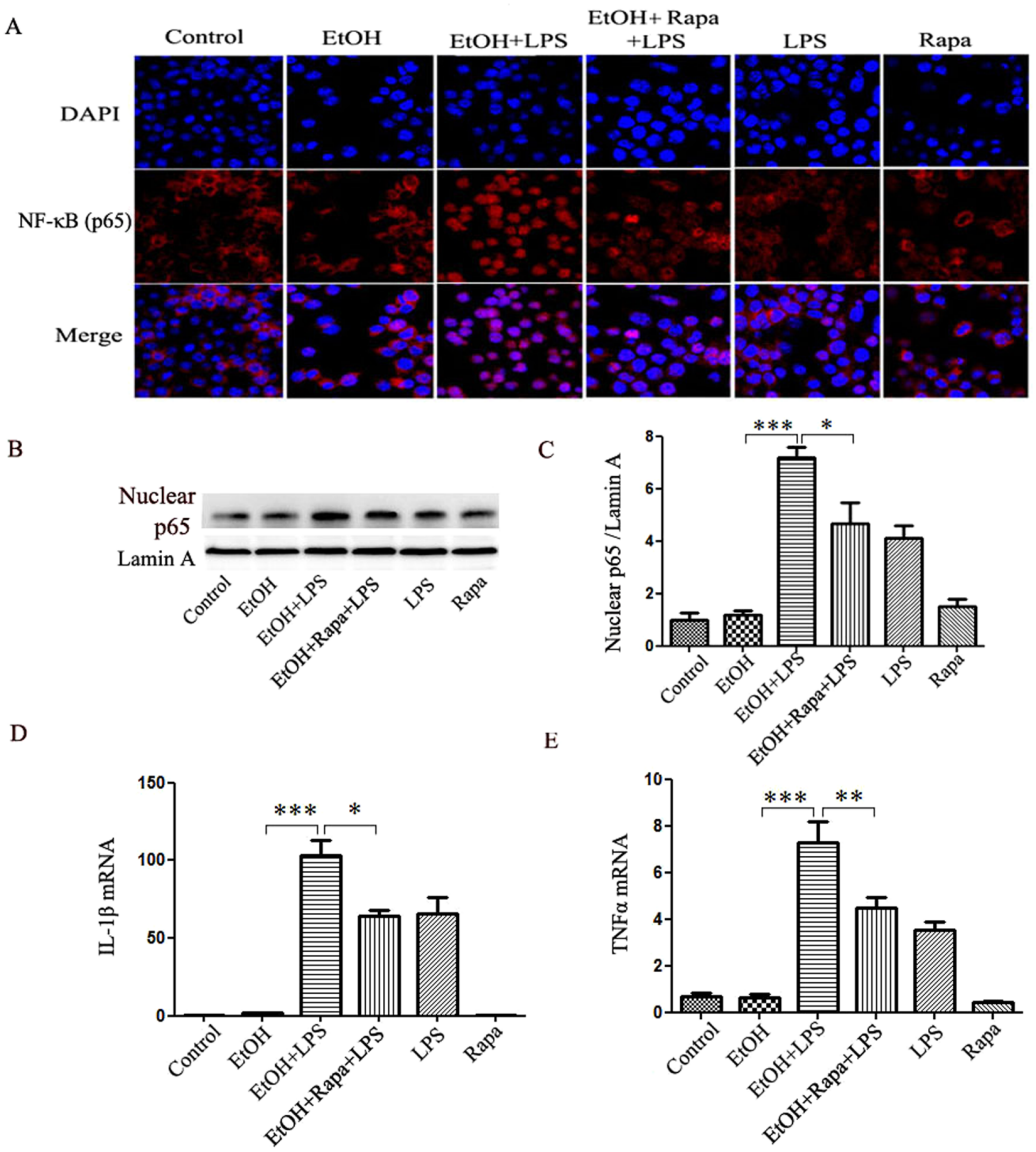
Induction of autophagy by rapamycin decreases p65 nuclear translocation and the production of pro-inflammatory cytokine. Raw264.7 cells were treated by alcohol and LPS in the presence or absence of rapamycin. (**A**) p65 (green) immunofluorescence staining. (**B**) Nuclear protein p65 levels. (**C**) Quantification analysis of p65 normalized to Lamin A levels. mRNA expression of IL-1β (**D**) and TNFα (**E**) in Raw264.7 cells. Data are expressed as mean ± SEM (n = 5). *p < 0.05. **p < 0.01, ***p < 0.001. Abbreviation: IL-1β, interleukin il- β; TNFα, tumor necrosis factor α.

We further examined the TLR4/MD2 mediated pro-inflammatory response by autophagy induction in EtOH-LPS treated Raw264.7 cells. Immunofluorescence staining showed a limited NF-κB p65 nuclear translocation induced by alcohol, but a significantly increased p65 nuclear level induced by EtOH-LPS treatment. This enhanced p65 nuclear translocation was markedly inhibited by rapamycin treatment (Fig. 7A). Confirming the results, Western blot analysis showed that nuclear p65 protein levels was increased by EtOH-LPS, but decreased by rapamycin treatment (Fig. 7B,C). As a consequence, IL-1β and TNF-α mRNA expression was inhibited by rapamycin (Fig. 7D,E).

**Figure 8:**
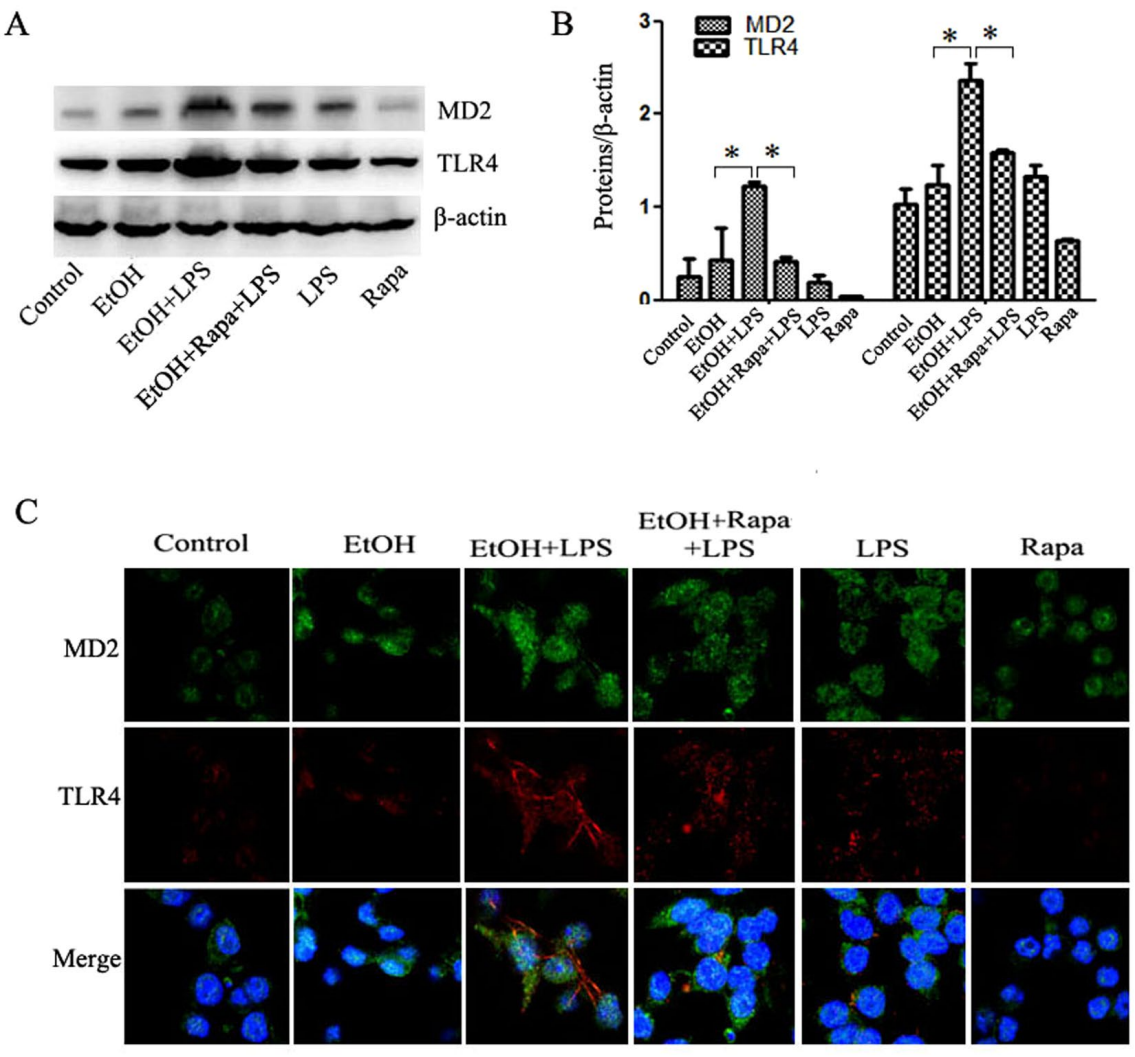
Rapamycin markedly attenuates expression and interaction of MD2 and TLR4. Raw264.7 cells were treated by alcohol for 48 hours followed by LPS for 6 hours. Rapamycin was added 24 h after alcohol treatment. (**A**) Expression of MD2 and TLR4 protein were detected by Western blotting. (**B**) MD2 and TLR4 expression was quantified by densitometry analysis and normalized to β-actin levels. (**C**) Immunofluorescent staining of MD2 (green) and TLR4 (red) in Raw264.7 cells. Images are at 1200 × magnification. Data are expressed as mean ± SEM (n = 5). *p < 0.05. Abbreviation: MD-2, myeloid differentiation 2; TLR, toll like receptor.

How do EtOH-LPS and rapamycin regulate the pro-inflammatory response in Raw264.7 macrophage cells? Protein levels of TLR4 and MD2 were dramatically increased by EtOH plus LPS treatment, which was reduced by autophagy induction by rapamycin as assessed by Western blotting and immunofluorescence detection (Fig. 8A–C). Importantly, EtOH + LPS treatment resulted in a co-localization of MD2 and TLR4, which was reduced by rapamycin.

Taken together, these findings suggest that hepatic macrophage cells are likely responsible for the beneficial effects of hepatic autophagy induction in livers exposed to EtOH and LPS by blocking MD2/TLR4 -mediated inflammatory signaling.

## Discussion

This study demonstrates that alcohol pre-exposure sensitizes the liver to injury from LPS through reduction of the autophagic response, and the induction of autophagy by rapamycin protects the liver from EtOH-LPS-induced fat accumulation and injury. We further demonstrate that the macrophage is responsible for the protective effect through inactivation of TLR4/MD2-mediated oxidative stress and inflammatory signaling. Although previous studies have demonstrated that autophagy induction has a beneficial function in ALD, the underlying mechanisms are not fully understood. Autophagy-mediated inhibition of TLR4/MD2 signaling may represent a molecular mechanism by which autophagy inducers decrease alcohol- and LPS-induced liver fat accumulation and injury.

Autophagy induction by alcohol depends on the exposure pattern in animal models^12, 13^. Previous studies demonstrated that acute alcohol ingestion activates autophagy, protecting against alcohol-induced steatosis and liver injury by removal of damaged/excess organelles such as mitochondria (mitophagy) and lipid droplets (lipophagy). In contrast, chronic alcohol exposure disrupts the hepatic autophagy response or slows autophagosome formation resulting in an increased steatosis^16^. In addition, the number of hepatic autophagosome/autolysome were slightly increased^21^ in a recently established binge-on-chronic alcohol exposure mouse model, which is quite common in human alcoholic^22^. A recent study further showed that this binge-on-chronic alcohol exposure increased mitophagy^23^.

Chronic alcohol-induced intestinal dysbiosis has been recognized as an important causative factor for the progression of ALD^24, 25^. Long term alcohol exposure significantly alters gut flora leading to increased gut barrier permeability^26^ and bacteria/bacterial products in the circulation^26, 27^, which in turn, activate TLR-mediated hepatic inflammation and steatosis^28^. Therefore, it is likely that the increased dysbiosis and LPS induced by chronic alcohol exposure may serve as a “second hit” that slows autophagy formation and damages the autophagic response to fat accumulation and inflammation. To test this hypothesis, we exposed mice to alcohol for 10 days followed by one dose LPS injection and examined the hepatic autophagy activation.

Confirming a previous report, we showed that 10-day alcohol feeding increased hepatic autophagy induction. It is likely that increased alcohol metabolism results in an elevated oxidant production which enhances autophagy acceleration. However, this alcohol pre-exposure primes hepatic cells to LPS-induced damage. This agrees with previous studies demonstrating that alcohol primes macrophages and increases LPS-induced pro-inflammatory cytokine production and leads to the development of ALD in a mouse model. Further studies showed that alcohol synergizes with LPS to upregulate the induction of TNF gene expression by decreasing cellular cAMP levels in macrophages^29^.

How does alcohol exposure prime liver for LPS insult? Studies have indicated that ROS production modulates autophagy, likely acting at multiple levels^30^. Ethanol-induced ROS serves as alarm molecules by presenting them to the autophagic machinery, which removes oxidative damage in a feedback manner. Therefore, it is likely that autophagic response by 10-day alcohol feeding does not attributed to the sensitization effect to LPS. Accumulating studies have demonstrated that oxidative stress derived from Cyp2e1 induction by alcohol sensitizes hepatocytes^31^ and Kupffer/macrophage^32, 33^ to LPS, leading to increased proinflammatory cytokine expression. In agreement with these observations, the current study clearly showed an increased NF-κB activation in the liver treated with alcohol and LPS.

Interestingly, LPS administration decreased the alcohol pre-exposure-induced autophagic response, leading to increased oxidative stress and inflammation in the liver. The mechanism by which alcohol pre-exposure functions together with LPS administration in reducing autophagic response is yet to know. However, activation of autophagy by rapamycin inhibited LPS-induced hepatic fat accumulation and inflammation in alcohol pre-exposed mouse liver.

How does rapamycin-activated autophagy function in EtOH-LPS-induced ALD? It is well-known that LPS binds to TLR4 to exert its pro-inflammatory function. TLR4 presents on the cell surface and is expressed mainly in the resident hepatic macrophages, Kupffer cells. The activation of TLR4 involves several components, including CD14 and MD2^34^. In particular, MD2 is associated with TLR4 and enhances the TLR4 response to LPS, since TLR4 itself does not response to LPS. The present study demonstrated that an LPS insult to the alcohol-primed macrophages induced a marked upregulation of both TLR and MD2 protein levels, and this increase was inhibited by rapamycin pretreatment. These results suggest that autophagy induction inhibits TLR4-mediated detrimental effects in the inflammatory response and oxidative stress in LPS-treated, alcohol-sensitized macrophages through decreasing TLR4 and MD2 protein expression and interaction.

In summary, we have demonstrated that alcohol pre-exposure sensitized the liver to LPS-induced hepatic inflammation and steatosis through dysregulation of LC3II-related autophagosome formation. Activation of autophagy by rapamycin attenuated EtOH-LPS-induced liver steatosis and injury by reducing TLR4/MD2 expression and interaction in macrophages. Our findings provided evidence to support the possible detrimental role of alcohol in priming macrophages to LPS-induced inflammatory response in ALD, which may help to better design pharmacological intervention against ALD targeting autophagy.

## Materials and Methods

### Animal experiments

Thirty-six male C57BL6 mice (8–10-wk of age, 25–30 g) were obtained from Harlan (Indianapolis, IN). They were maintained at 22 °C with a 12 h light/dark cycle and had free access to normal chow diet and tap water. Each group of mice was initially fed the control Lieber-DeCarli liquid diet (Bio-Serve, Flemingtown, NJ) for 3 days to acclimate the mice to the liquid diet. Afterward, a half of the mice were fed the Lieber-DeCarli liquid alcohol diet (alcohol-fed, AF) and the other half of mice were fed the control isocaloric liquid diet (alcohol was replaced by maltose dextrin, pair-fed, PF). The content of alcohol in the liquid diet was gradually increased from 1.6% (w/v) to 5% (w/v) in first 6 days, and remained at 5% for subsequent 10 days. LPS injection at a dose of 10 mg/kg via i.p was conducted on the last day in the morning. Six hours later, the mice were sacrificed for analysis. In one group, rapamycin was administered at a dose of 2 mg/kg one day before LPS injection. At the end of the experiment, the mice were anesthetized with Avertin. Plasma and tissue samples were collected for assays. All mice were treated according to the protocols reviewed and approved by the Institutional Animal Care and Use Committee of the University of Louisville, and all the procedures were carried out in accordance with the approved guidelines.

### Cell culture

RAW264.7 cells (mouse macrophage cell line) were cultured in Dulbecco’s modified Eagle’s medium (DMEM, Invitrogen) containing 10% fetal bovine serum and 1% penicillin/streptomycin, and were maintained at 37 °C and 5% CO_2_. RAW264.7 cells were utilized for experimentation at 70–80% confluency. The cells were exposed to varying doses of alcohol for 48 hours prior to the treatment with LPS (Escherichia coli 0111: B4, Sigma-Aldrich, St. Louis) at 100 ng/mL for 6 hours. Cell viability was not affected by ethanol or LPS treatment at the doses used in the experiments. Rapamycin was administered (50 nmol/L) one day before LPS treatment.

### Liver injury and lipid accumulation

Hepatic fat accumulation and liver injury were evaluated by hepatic tissue hematoxylin and eosin (HE) staining, Oil red O staining, liver tissue triglyceride (TG) levels, serum levels of alanine aminotransferase (ALT) and aspartate aminotransferase (AST). Formalin-fixed paraffin tissue sections were processed for HE staining and frozen tissue sections were processed for Oil red O staining. Liver TG levels were determined using a triglyceride kit (Thermo Scientific, Waltham, MA). Serum ALT or AST activities were measured using ALT and AST assay kit (Thermo Scientific) according to the manufacturer’s instructions.

### ROS determination

Reactive oxygen species (ROS) accumulation in the liver was examined by dihydroethidium (DHE) staining. Nonfluorescent dihydroethidium is oxidized by ROS to yield the red fluorescent product, ethidium that binds to nucleic acids and stains the nucleus with bright fluorescent red. Cryostat sections of liver were incubated with 5μmol/L DHE (Molecular Probes, Eugene, OR) for 30 min at 37 °C in the dark. The ROS-catalyzed ethidium red fluorescence was examined under confocal microscopy. The relative fluorescence intensity was quantified using Sigma Scan Pro 5 software.

### Real time PCR assay

The mRNA levels were assessed by real-time PCR. In brief, the total RNA was extracted with Trizol according to manufacturer’s protocol (Invitrogen, Carlsbad, CA) and reverse-transcribed using GenAmp RNA PCR kit (Applied Biosystems, Foster City, CA). Quantitative real-time PCR was performed on an ABI 7500 real-time PCR thermocycler, whereas SYBR green PCR Master Mix (Applied Biosystems) was used for real-time PCR analysis. The relative quantities of target transcripts were calculated from duplicate samples after normalization of the data against the housekeeping gene, β-actin. Dissociation curve analysis was performed after PCR amplification to confirm the specificity of the primers. Relative mRNA expression was calculated using the Ct method. The following primer pairs were used: IL-1β forward 5′TTCATCTTTGAAGAAGAGCCCAT3′, reverse 5′TCGGAGCCTGTAGTGCAGTT3′; IL-6 forward 5′TGGAAATGAGAAAAGAGTTGTGC3′, reverse 5′CCAGTTTGGTAGCATCCATCA3′; TNFα forward 5′CCAGCCGATGGGTTGTACCT3′, reverse 5′TGACGGCAGAGAGGAGGTTG3′.

### Western blot

Hepatic tissues or cultured cells were lysed in immunoprecipitation buffer containing 50 mM Tris-HCl, 150 mM NaCl, 5 mM MgCl_2_, 2 mM EDTA, 1 mM NaF, 1% NP-40, and 0.1% sodium dodecyl sulfate, and centrifuged for 15 min at 12000 g at 4 °C. The nuclear extract was prepared according to the manufacturer’s instruction using NE-PER nuclear and cytoplasmic extracts reagents kits (Pierce, Rockford, IL). The protein concentration was measured using a BCA assay kit (Pierce, Rockford, IL). Sample aliquots (usually 100 μg) were boiled for 5 min and equal protein amounts (usually 30 μg) were separated by 10% SDS-PAGE. Proteins were then transferred to a nitrocellulose membrane. Blots were blocked and immunoblotted with anti-Cyp2E1 (ab28146), NF-κB p65 (sc-372), IκBα (sc-1643), Histone (ab1791), Beclin-1(ab55878), LC3 (ab128025), lamin A (ab26300), MD2 (ab24182), TLR4 (sc-293072) and β-actin (sc-81178) at 4 °C overnight, followed by HRP-conjugated secondary antibodies (typically 1:2000 dilution) for 1 h at room temperature. The results were analyzed with Gel-Pro Image Analysis Software.

### Immunofluorescence

After deparaffinization, serum blocking and antigen retrieval, tissue sections underwent immunofluorescence staining with primary antibodies against p65 (1:200), LC3 (1:200), MD2 (1:200) and TLR4 (1:200) at 4 °C overnight. Alexa Fluor 488- or 594-conjugated secondary antibodies (Molecular Probes, Carlsbad, CA) were added to sections for 1 h at room temperature. DAPI was used for nuclear counterstaining. Sections were mounted with a coverslip, sealed with nail polish and stored in the dark at 4 °C.

Raw264.7 cells were cultured on glass slides, washed in PBS and fixed with 4% paraformaldehyde for 15 min at room temperature. Cells were then permeabilized, blocked with 1% BSA and incubated with primary and secondary antibodies. Cells were co-stained with DAPI for the nuclei. Images were captured from six or more randomly chosen fields using a laser scanning confocal microscope. Data from repeated experiments are subjected to statistical analysis.

### Statistics

All data are expressed as means ± SEM at least three experiments. The data were analyzed by ANOVA and Newman-Keuls multiple-comparison test. A P value < 0.05 was considered to indicate a statistically significant difference.

## Electronic supplementary material

Supplementary information
