# Supplementary material for: Activation of autophagy attenuates EtOH-LPS-induced hepatic steatosis and injury through MD2 associated TLR4 signaling
Source: Sci Rep. 2017 Aug 24;7:9292. doi: 10.1038/s41598-017-09045-z (PMC5571015; doi:10.1038/s41598-017-09045-z)
Supplement: Supplementary file 1 — Supplementary information [file 41598_2017_9045_MOESM1_ESM.pdf]

# Activation of autophagy attenuates EtOH-LPS-induced hepatic steatosis and injury through MD2 associated TLR4 signaling

Xiaoxia Kong[1,4,*], Ying Yang[2,4], Li Ren[3,4], Tuo Shao[4], Fengyuan Li[4], Cuiqing Zhao[2,4,5], Liming Liu[4] , Hongyu Zhang[2], Craig J. McClain[4,7], Wenke Feng[2,4,5,6,*]

[1]School of Basic Medical Sciences. [2]School of Pharmaceutical Sciences, Wenzhou Medical University, Wenzhou, Zhejiang, China. [3]1st Affiliate Hospital, Xi'an Jiaotong University, Xi'an, China. [4]Departments of Pharmacology and Toxicology and Medicine, University of Louisville Alcohol Research Center, University of Louisville Hepatobiology & Toxicology Program, University of Louisville, Louisville, USA. [5] Institute of Virology, Wenzhou University, [6] 2nd Affiliate Hospital, Wenzhou Medical University, [7]Robley Rex Louisville VAMC, Louisville, KY, USA.

Correspondence should be addressed to W.F. (email: wenke.feng@louisville.edu) or X.K. (email: kongxx@wmu.edu.cn)

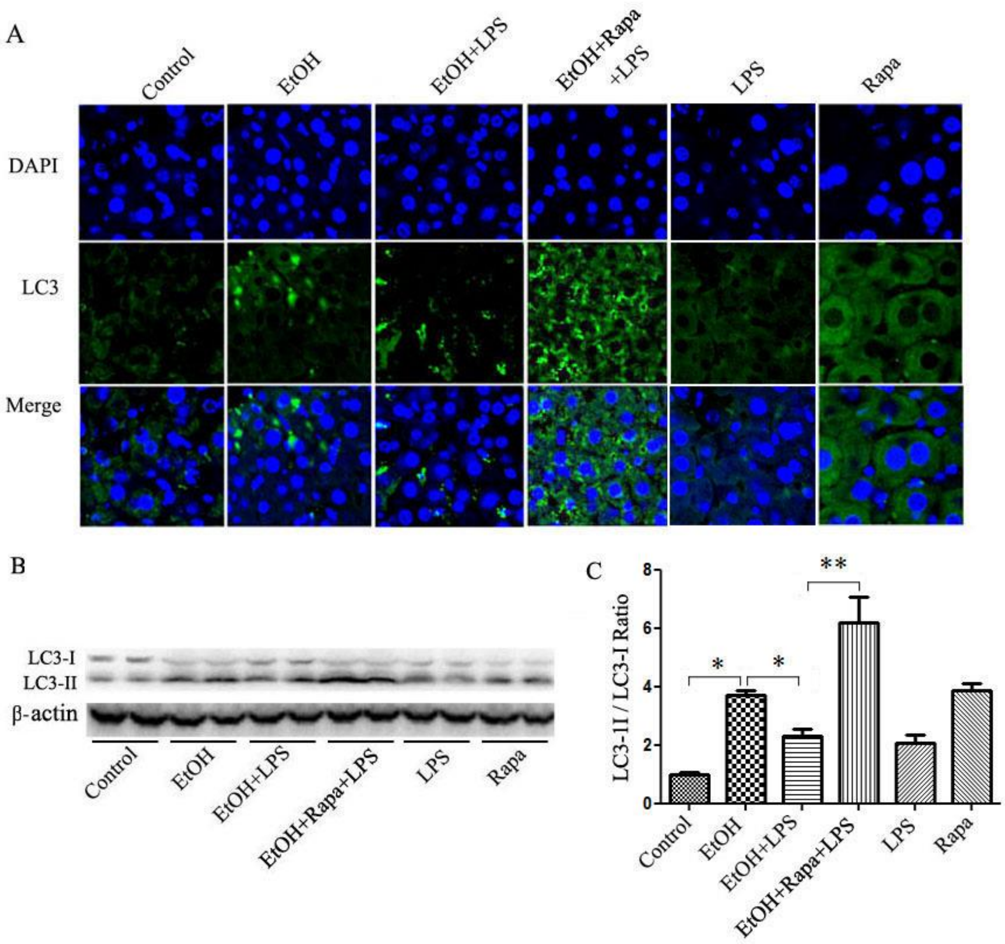


**Supplementary Figure S1.** Rapamycin activates hepatic autophagy in the liver exposed to EtOH+LPS. (A) Immunostaining analysis of LC3 (green). (B) Expression of LC3 protein detected by Western blotting. (C) LC3 expression was quantified by densitometry analysis and normalized to β-actin expression. Data are expressed as mean ± SEM (n=6). $^*p < 0.05$, $^{**}p < 0.01$. Abbreviation: LC3, microtubules associated protein 1 light chain 3β.

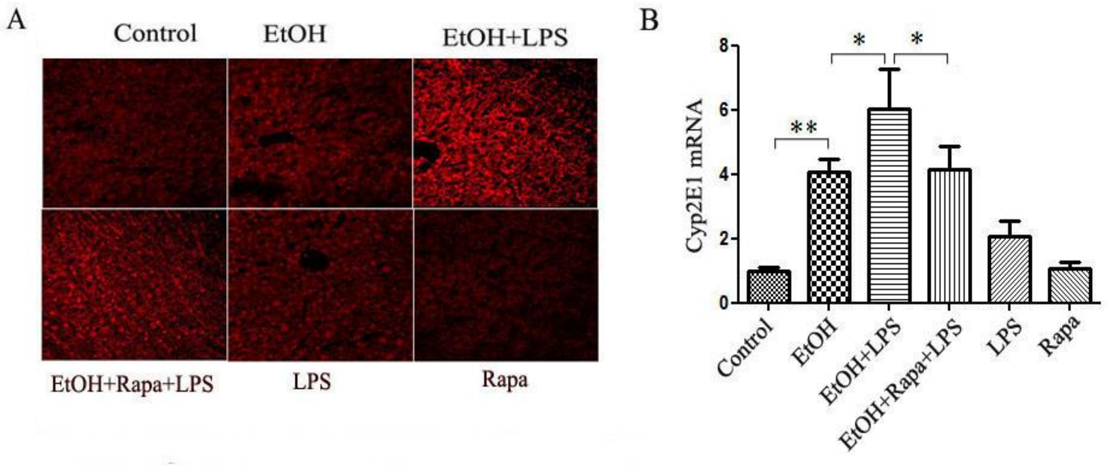


**Supplementary Figure S2.** Rapamycin decreases hepatic ROS formation. (A) DHE staining of frozen liver sections. (B) Liver Cyp2E1 mRNA levels. Data are expressed as mean ± SEM (n=6). *p < 0.05, **p < 0.01. Abbreviation: DHE, dihydroethidium; Cyp2E1, cytochromeP450.

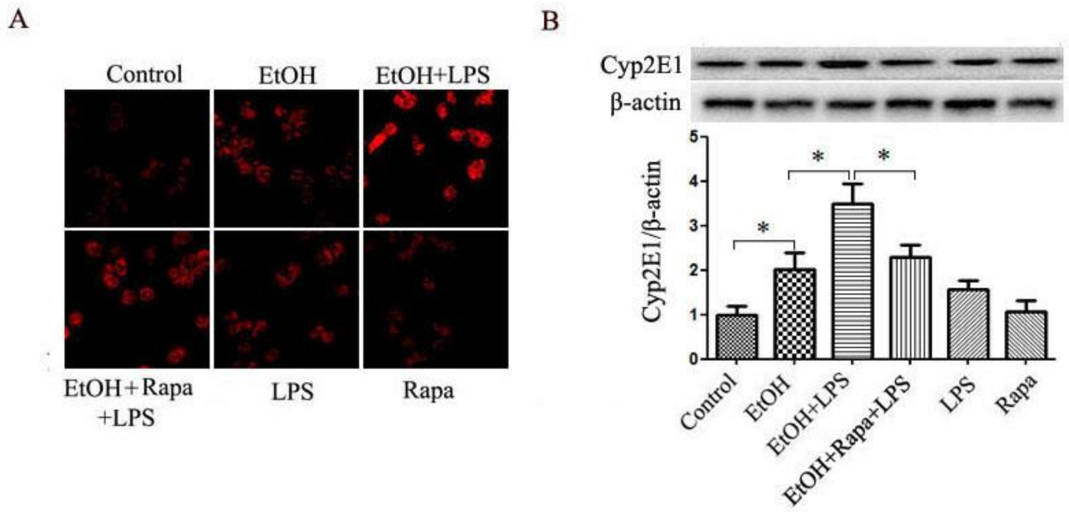


**Supplementary Figure S3.** Induction of autophagy by rapamycin decreases ROS formation in Raw264.7 cells treated with EtOH+LPS. (A) DHE staining of cells. (B) Liver Cyp2E1 protein level was analyzed by Western blotting and the ratio of Cyp2E1 to β-actin was calculated. Bars represent mean ± SEM, (n=5). *p < 0.05. Abbreviation: DHE, dihydroethidium; Cyp2E1, cytochromeP450.

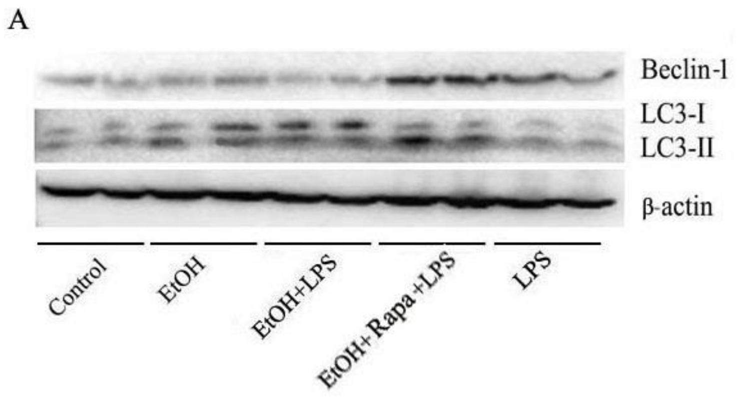


**Supplementary Figure S4. Rapamycin increases autophagy activation by EtOH-LPS.** Mice were fed 5% alcohol in the liquid diet for 10 days. LPS injection at a dose of 10 mg/kg via i.p was conducted on the last day in the morning. Six hours later, the mice were sacrificed for analysis. In one group, rapamycin was administered at a dose of 2 mg/kg one day before LPS injection. (**A**) Expression of Beclin-1 and LC-3 proteins in the liver tissue were detected by Western blotting.
